# Oligomeric State and Thermal Stability of Apo- and Holo- Human Ornithine δ-Aminotransferase

**DOI:** 10.1007/s10930-017-9710-5

**Published:** 2017-03-27

**Authors:** Riccardo Montioli, Carlotta Zamparelli, Carla Borri Voltattorni, Barbara Cellini

**Affiliations:** 1 Department of Neuroscience, Biomedicine and Movement Sciences (Section of Biological Chemistry), University of Verona, Strada Le Grazie 8, 37134 Verona, Italy; 2 Department of Biochemical Sciences, University “La Sapienza”, Rome, Italy

**Keywords:** Pyridoxal 5′-phosphate, Tetramer–dimer equilibrium, Interface contacts, Protein stability, Coenzyme, Quaternary structure

## Abstract

Human ornithine δ-aminotransferase (hOAT) (EC 2.6.1.13) is a mitochondrial pyridoxal 5′-phosphate (PLP)-dependent aminotransferase whose deficit is associated with gyrate atrophy, a rare autosomal recessive disorder causing progressive blindness and chorioretinal degeneration. Here, both the apo- and holo-form of recombinant hOAT were characterized by means of spectroscopic, kinetic, chromatographic and computational techniques. The results indicate that apo and holo-hOAT (a) show a similar tertiary structure, even if apo displays a more pronounced exposure of hydrophobic patches, (b) exhibit a tetrameric structure with a tetramer-dimer equilibrium dissociation constant about fivefold higher for the apoform with respect to the holoform, and (c) have apparent T_m_ values of 46 and 67 °C, respectively. Moreover, unlike holo-hOAT, apo-hOAT is prone to unfolding and aggregation under physiological conditions. We also identified Arg217 as an important hot-spot at the dimer–dimer interface of hOAT and demonstrated that the artificial dimeric variant R217A exhibits spectroscopic properties, T_m_ values and catalytic features similar to those of the tetrameric species. This finding indicates that the catalytic unit of hOAT is the dimer. However, under physiological conditions the apo-tetramer is slightly less prone to unfolding and aggregation than the apo-dimer. The possible implications of the data for the intracellular stability and regulation of hOAT are discussed.

**Electronic supplementary material:**

The online version of this article (doi:10.1007/s10930-017-9710-5) contains supplementary material, which is available to authorized users.

## Introduction

Ornithine δ-aminotransferase (OAT) (EC 2.6.1.13), or ornithine: 2-oxo-glutarate aminotransferase, is a pyridoxal 5′phosphate (PLP)-dependent enzyme that catalyzes the δ-transamination of l-ornithine (l-Orn) and α-ketoglutarate (α-KG) to glutamic-γ-semialdehyde (GSA) and l-glutamate in the mitochondrial matrix. GSA then spontaneously cyclizes forming pirroline-5-carboxylate (P5C), a proline precursor. Mammalian OAT is mostly present in liver, kidney and eyes, even if the enzyme is expressed in almost all tissues [1–3]. The proposed physiological role of OAT is to control the intra-mitochondrial concentration of l-Orn. Recently, a role for OAT in mitotic cell division has been proposed, suggesting that the protein could represent a target for chemotherapeutic drug development [4]. The enzyme is also of medical interest because the OAT deficit causes gyrate atrophy, a rare autosomal recessive hereditary disorder leading to progressive blindness and chorioretinal degeneration characterized by the appearance of sharply demarcated circular areas of chorioretinal atrophy [5, 6].

OAT is synthesized as a 49 kDa precursor in the cytosol and it is then imported into mitochondria where it reaches the functional conformation upon removal of an N-terminal mitochondrial targeting sequence (residues 1–25) producing a ~45 kDa mature protein [7]. Unlike OATs from plant sources that were found monomeric [8, 9] or from some bacterial [10] or protozoa [11, 12] that were found homodimeric, mammalian OATs show a higher level of subunits assembly but their quaternary structure is controversial. In fact ultracentrifugation and SEC studies on the OAT purified from rat and human liver [13–15] indicated that the enzyme has a tetrameric assembly. Accordingly, high resolution electron microscopy studies on OAT purified from pig kidney indicated that the holoenzyme is a homotetramer [16]. Moreover, Sanada et al. have reported that the apo-OAT exists in dimeric form [14] raising a question about the possible role of the PLP in the OAT oligomerization. Nevertheless, analytical ultracentrifugation studies on crude extract of rat liver have suggested that OAT presents a molecular weight dependent on the enzyme concentration assuming a two-step aggregation of the monomer forming at first trimers (130–140 kDa) and then hexamers (280 kDa) [17]. Crystallographic studies on purified rat liver OAT [18] and, more recently, on human recombinant OAT (hOAT) both ligand-free [19] and in complex with different substrate analogues [20, 21], indicated that the human enzyme could also assume an hexameric assembly composed by three homodimers hold together mainly by electrostatic interactions. Moreover, on the basis of the hOAT crystal structure, the dimeric unit of the enzyme belongs to the fold type I class of PLP-enzymes and it was proposed that the dimer could represent the functional unit of the enzyme [19]. However, a comprehensive investigation of the biochemical properties of the hOAT as well as of the coenzyme role on hOAT oligomerization and stability is still lacking. Here, we demonstrated that although both holo- and apo-hOAT share a similar tertiary structure and a tetrameric structure, the apo, as compared with the holo, exhibits (a) a more pronounced exposure of hydrophobic surface, (b) a fivefold higher tetramer–dimer equilibrium dissociation constant (K_D(tet−dim)_) value, (c) a 21 °C lower T_m_ value, and (d) an higher propensity to aggregation under physiological conditions. Additionally, the identification of Arg217 as a hot spot of dimer–dimer interface prompted us to construct, clone, and purify the R217A mutant. Its biochemical characterization revealed that the mutant form has a dimeric structure with spectroscopic and catalytic features as well as apparent T_m_ values comparable with the corresponding ones of tetrameric hOAT, thus strongly suggesting that in hOAT the dimer is the functional unit. Nevertheless, we found that the apo-tetrameric form is slightly less prone to unfolding and aggregation than the apo-dimeric one. Based on these results, the possible role of PLP in the intracellular stability and oligomeric state of hOAT is discussed.

## Materials and Methods

### Materials

PLP, l-Orn, α-KG, 2-aminobenzaldehyde, dimethyl sulphoxide (DMSO), isopropyl-β-d-thiogalactoside (IPTG), phenylhydrazine hydrochloride and phenylmethylsulfonyl fluoride (PMSF) were purchased from Sigma. 1,8-Anilino-naphthalene sulfonic acid (ANS) was purchased from Molecular Probes. All other chemicals were of the highest purity available. The pYES2.1::hOAT expression plasmid coding for the complete cDNA of hOAT [22] was kindly provided by Prof. Leonardo Salviati of the University of Padova (Italy).

### Cloning and Site-Directed Mutagenesis

The 26–439 coding sequence of hOAT was equipped with 5′ NdeI and 3′ *Hind*III restriction sites by extension PCR using the pYES2.1::hOAT vector as template and the 5′ CGGCTCATATGACATCTGTTGCAACTAAAAAAACAG 3′ forward primer and the 5′ CCGACAAGCTTTCAGAAAGACAAGATGGTCTTG 3′ reverse primer. To construct the pOAT expression plasmid, the amplified sequence was cloned in a pET43a expression vector by means of the above mentioned restriction sites. The pOAT-R217A expression vector was constructed using the Quick change II kit (Agilent Technologies), the pOAT vector as template, and the oligonucleotide 5′ CTGCCCGCACTGGAGGCTGCTCTTCAGGATCCAAATG 3′ and its complement. The mutation was confirmed by DNA sequence analysis of the whole coding region.

### Expression and Purification

*Escherichia coli* BL21(DE3)pLysS cells transformed with the pOAT or pOAT-R217A mutant expression plasmid were grown in 4.5 L of Luria broth at 37 °C to a turbidity of 0.6–0.8 at 600 nm. Expression was induced with 0.5 mM IPTG for 15 h at 30 °C. Cells were harvested and resuspended in 20 mM sodium phosphate buffer, pH 7.6 (buffer A) containing a protease inhibitor cocktail (Roche), 0.1 mM EDTA, 50 μM PLP and 0.5 mM PMSF. Lysozyme was added to a concentration of 0.2 mg/mL and the culture was incubated for 20 min at room temperature. After a freeze–thaw, leupeptin (0.5 μg/mL) and pepstatin (0.7 μg/mL) were added and the suspension was centrifuged at 30,000*g* for 30 min at 4 °C. The supernatant was recovered, adjusted to pH 7.6 and loaded on a DEAE Sepharose 26/20 equilibrated with buffer A. A linear gradient (240 mL) from 20 to 150 mM sodium phosphate buffer, pH 7.6, was then applied. Under these conditions, OAT elutes from the column between 110 and 140 mM sodium phosphate. Active fractions were pooled and reconstituted with 100 μM PLP. The solution was then concentrated using an Amicon Ultra 15 unit (Millipore) and applied to a Superdex 200 XK 26/60 column (GE Healthcare) equilibrated in 50 mM Hepes, pH 7.4, NaCl 200 mM. The eluted protein was concentrated using an Amicon Ultra 15 unit and stored at −20 °C. This method, yielding about 35 mg of pure protein per litre of bacterial culture, is summarized in Table 1. The PLP content of the enzyme was determined by releasing the coenzyme in 0.1 M NaOH and by using ε_M_ = 6600 M^−1^ cm^−1^ at 388 nm. The apparent molar absorption coefficient at 280 nm of OAT dimer was determined following the method of Pace [23] and found to be 124,767 M^−1^ cm^−1^. In this work the enzymatic molar concentration is expressed as dimer equivalents.

**Table 1:** Purification procedure of hOAT from 1 L of bacterial culture

	Protein (mg)	Total activity (units)	Specific activity (units/mg)	Yield (%)	Purification (fold)
Cleared lysate	830	1950	2.3	100	–
DEAE sepharose	72	1220	17	62	7.4
Superdex 200	35	775	22	40	9.6

### Enzyme Activity Assay

The OAT enzymatic activity was determined by a spectrophotometric assay measuring the dihydroquinazolium derivative of P5C after incubation with 2-aminobenzaldehyde. The purified protein (0.06–1 μM) was incubated with saturating concentration of l-Orn (100 mM) and α-KG (50 mM) in 50 mM Hepes, pH 8.0, 150 mM NaCl at 25 °C in the presence of 100 μM PLP. Reaction was stopped by addition of trichloroacetic acid 10% (v/v). A fresh solution of o-aminobenzaldehyde was prepared in 0.7 M HCl, 30% DMSO, and added to the samples at 15 mM final concentration. Samples were incubated at 25 °C for 40 min to allow the development of the color. After centrifugation (12,000*g* for 2 min), the absorbance at 440 nm of the recovered supernatant was measured and a molar extinction coefficient of 2.71 × 10^3^ M^−1^ cm^−1^ was used to calculate the amount of P5C formed.

### Apoenzyme Preparation

The apo-form of wild-type OAT and of the R217A variant were obtained by incubating the enzyme at 5 μM concentration with 80 mM phenylhydrazine hydrochloride in 0.5 M potassium phosphate buffer pH 6.9 for 1 h at 25 °C in the dark. The solution was then loaded on a Hiprep 26/10 desalting column (Amersham) equilibrated with 50 mM Hepes, pH 7.4, 0.5 M NaCl. The eluted apoenzyme was concentrated by means of an Amicon ultra 4 unit (Millipore) and stored at −20 °C.

### Spectroscopic Measurements

Absorption measurements were performed by a Jasco V-550 spectrophotometer at a protein concentration of 6 μM. CD measurements were made by a Jasco J-710 spectropolarimeter at a protein concentration of 6 μM. The ANS emission spectra were recorded upon excitation at 365 nm of a 6 μM enzyme sample previously incubated with 150 μM ANS for 1 h at 25 °C in the dark. The ANS binding of the holo-forms was registered in the presence of 20 µM PLP. Measurements were performed by a FP Jasco spectrofluorimeter setting 5 nm excitation and emission bandwidths. Spectroscopic analyses under physiological conditions were performed in phosphate-buffered saline (PBS) pH 8.0. All other spectroscopic measurements were carried out in 50 mM Hepes, pH 7.4, 0.5 M NaCl. The equilibrium dissociation constant for PLP (K_D(PLP)_) of wild-type and the mutant enzyme was determined by measuring the quenching of the intrinsic fluorescence of the apoenzyme at 90 nM upon incubation with PLP at a concentration range of 0.01–10 μM for 2 h at 25 °C. The K_D(PLP)_ value was obtained using the following equation:

1 $$Y = Y_{{MAX}} \frac{{\left[ E \right]_{t} + \left[ {PLP} \right]_{t} + K_{{D\left( {PLP} \right)}} - \sqrt {\left( {\left[ E \right]_{t} + \left[ {PLP} \right]_{t} + K_{{D\left( {PLP} \right)}} } \right)^{2} - 4\left[ E \right]_{{t}} \left[ {PLP} \right]_{t} } }}{{2\left[ E \right]_{t} }}$$

where [E]_t_ and [PLP]_t_ represent the total concentration of OAT and PLP, respectively, Y refers to the changes of the emission fluorescence signal at 338 nm at a PLP concentration [PLP]_t_, and Y_max_ refers to the maximum change of the fluorescence intensity when all enzyme molecules are complexed with coenzyme.

### In Silico Analysis

The OAT tetramer was constructed starting from the available pdb file of hOAT (1OAT.pdb). The structures of the complete dimer were used as template to add the missing chain by means of the structural alignment tool of the MOE software (Chemical Computing Group) [24]. The protonation state was applied by PROTONATE 3D tool of MOE software setting 150 mM ionic strength and pH 8.0. The structure was refined using the energy minimization tool of the MOE software applying the AMBER99 force field.

### Dynamic Light Scattering (DLS) Analysis

DLS measurements were made on a Zetasizer Nano S device from Malvern Instruments. To analyze the particles diameter, the enzymatic species were diluted in 50 mM Hepes, pH 7.4, 0.5 M NaCl at 25 °C. PLP was added to the holoenzyme solutions to a final concentration of 50 μM. The buffer was filtered using Anotop 10 filters (Whatman) immediately before use to eliminate any impurities.

### Analytical Ultracentrifugation (AUC)

Sedimentation velocity experiments were performed on a Beckman Coulter Proteomelab XLI analytical ultracentrifuge equipped with absorbance optics. The experiments were conducted at 25,000 rpm at 20 °C at a protein concentration of 6 µM in the buffers 50 mM Hepes 0.5 M NaCl pH 7.4. Radial absorbance scans were collected at 280 nm at a spacing of 0.003 cm with three average in a continous scan mode. Sedimentation coefficients were determined using the program Sedfit (provided by Dr P. Schuck, National Institutes of Health) and were reduced to water (S_20,W_) using standard procedures.

### Size Exclusion Chromatography (SEC) Analysis

SEC experiments were performed with an AKTA FPLC system (GE Healthcare) equipped with an UV–visible detector at 280 nm using a custom packed Sephacryl S-300 10/600 column. The calibration curve of the column was obtained by means of gel filtration standard markers (Bio-Rad) and resulted in:

$${\text{Log(MW)}} = - 0.{\text{113Ve}} + 8.22$$

where MW is the molecular weight and Ve is the elution volume of the protein. Apo and holo-hOAT samples were dissolved in running buffer at different concentrations (ranging from 0.3 to 75 μM) and incubated for 30 min at 25 °C. Samples were then loaded on the column equilibrated in 50 mM Hepes, pH 7.4, 0.5 M NaCl. The injection volume was 0.1 mL and the flow rate was 0.4 mL/min. The elution profiles were analysed using the software Unicorn 5.0 (GE Healthcare). The percentage of tetramer (%T) of each sample was calculated from the elution curves following the method of Manning et al. [25]. Assuming that the elution volume (Ve) varies as a function of the molecular weight (MW) it follows that:

2 $${\text{Log}}\left( {\text{2}} \right)/\left( {{\text{Vd}} - {\text{Vt}}} \right) = {\text{Log}}\left( {{\text{MW}}/{\text{D}}} \right)/\left( {{\text{Vd}} - {\text{Ve}}} \right),$$

where D is the molecular weight of the dimer, Vd and Vt are the elution volumes of the dimeric and tetrameric species respectively. At a given enzyme concentration the effective MW is given by:

3 $${\text{MW}} = {\text{D}}\left( {{\text{1}} + \% {\text{T}}/{\text{1}}00} \right),$$

where D is the MW of the dimeric species and %T is the percentage of the tetramer present. Combining Eqs. 2 and 3 gives:

4 $$\% {\text{T}} = {\text{1}}00\left( {{\text{2}}^{{({\text{Vd}} - {\text{V}})/({\text{Vd}} - {\text{Vt}})}} - {\text{1}}} \right)$$

The dimer–tetramer equilibrium dissociation constant (K_D(dim−tet)_) was calculated following the method of Manning et al. [25]. Assuming that [T_tot_] is the maximal amount of OAT tetramer and [T] and [D] denote the concentrations of tetrameric and dimeric OAT respectively, the plot of log(%T/0.04 (100 − %T)^2^) vs. log [T_tot_], gives a straight line with a slope 1. When (%T/0.04 (100 − %T)^2^) = 1, K_D(dim−tex)_ = [T_tot_].

## Results

### Solubility of Purified Recombinant hOAT

hOAT frozen at −20 °C at a concentration higher than 200 µM in phosphate buffer, forms visible aggregates after thawing. On the other hand, apo-hOAT dissolved in phosphate buffer is prone to aggregation. In particular, at physiological ionic strength it gives rise to visible aggregates. Thus, we checked the solubility of apo- and holo-hOAT in different buffers and at different ionic strength conditions by measuring their propensity to aggregation as well as the loss of catalytic activity after storage at −20 °C. We found that 50 mM Hepes containing 500 mM NaCl, pH 7.4, was the buffer that maintained the solubility of both forms of hOAT. For this reason, the analyses comparing the spectroscopic properties and the apparent T_m_ values of apo and holo were carried out under these experimental conditions.

### Spectral and Kinetic Properties of hOAT

**Fig. 1:**
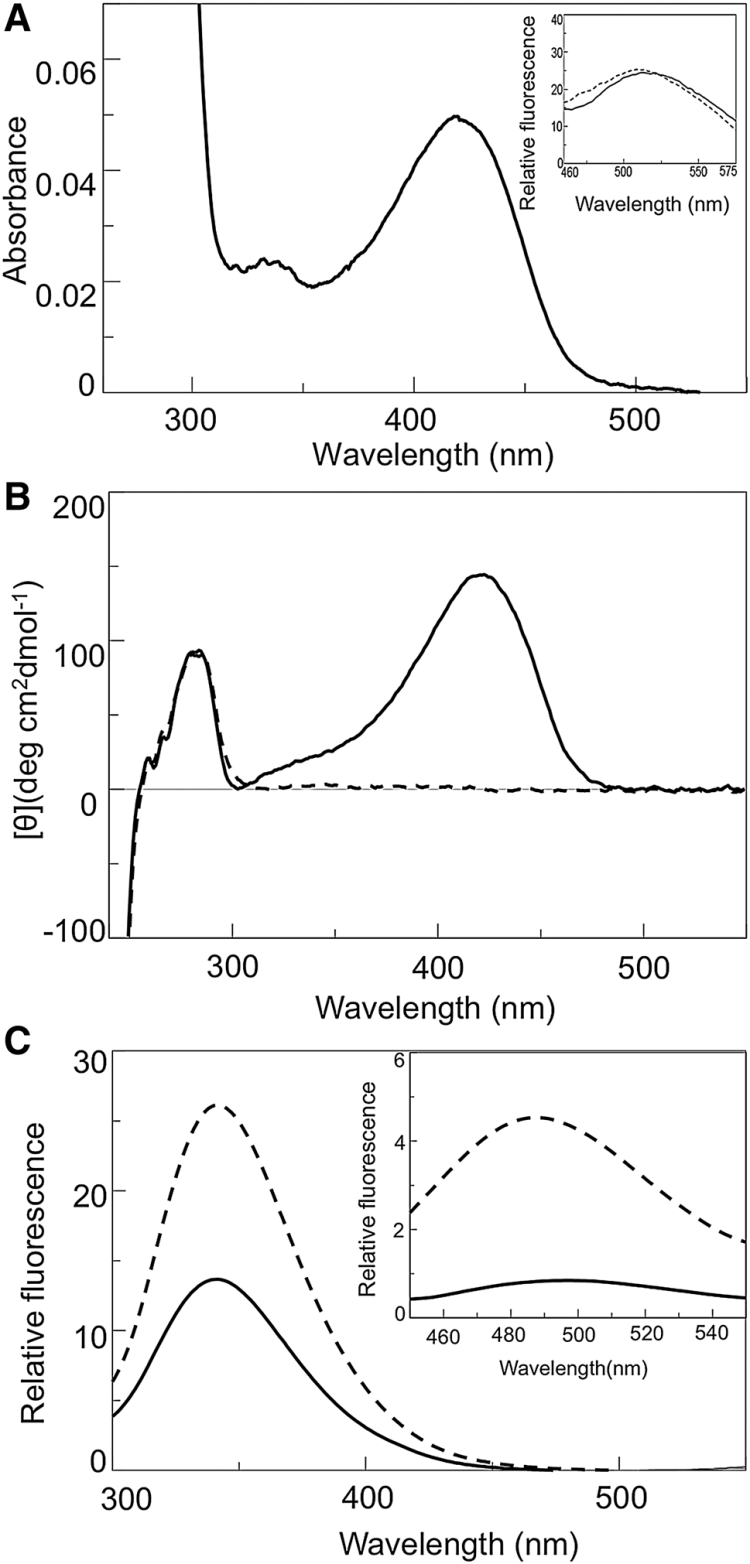
Spectroscopic features of wild-type hOAT. **a** Absorbance spectra of holo-hOAT; **b** CD and **c** fluorescence spectra of holo- (*normal line*) and apo- (*dashed line*) hOAT. The *inset* of **a** shows the coenzyme fluorescence upon excitation at 420 nm (*normal line*) and 340 nm (*dashed line*). The *inset* of **c** shows the ANS emission fluorescence spectra of holo- (*normal line*) and apo- (*dashed line*) hOAT. Spectra were registered at 6 μM enzyme concentration in 50 mM Hepes, pH 7.4, 0.5 M NaCl

Recombinant purified hOAT bind ~2 moles of PLP/mol of dimer. The deconvolution of the far-UV CD spectrum of the enzyme at 0.3 μM concentration gives a secondary structure composition of 37% α-helix, 18% β-sheet and 45% random coil, values identical to those calculated from the solved crystal structure [19]. While apo-hOAT does not show any absorbance or dichroic band in the visible region, holo-hOAT exhibits two bands at 420 and 340 nm corresponding to a positive CD band at 422 nm and a minor shoulder around 340 nm, respectively (Fig. 1a, b). The A_420_/A_340_ ratio is equal to ~3 in the pH range 6.0–9.0. As shown in the inset of Fig. 1a, upon excitation at 420 or at 340 nm, the fluorescence emission spectra of holo-hOAT exhibit a maximum around 505–510 nm, thus allowing us to attribute the 420 and 340 nm absorbance and CD bands to the ketoenaminic and enoliminic tautomeric forms of the PLP-Lys292 [19] internal aldimine, respectively. In the near-UV region the apo- and holo-forms of hOAT show a positive dichroic band at 284 nm of the same magnitude. Upon excitation at 280 nm (Fig. 1c), holo and apo-hOAT exhibit an emission maximum at 341 and 342 nm, respectively. The emission intensity of apo-hOAT is roughly twofold higher than that of holo-hOAT, as a consequence of the quenching of the emission fluorescence due to the bound PLP in the holoform. The ANS emission spectrum of apo-hOAT exhibits an intensity tenfold higher and a maximum 5 nm blue-shifted as compared with that of holo-hOAT (inset of Fig. 1c), thus indicating that the apo-form exposes more hydrophobic patches with respect to the holo-form. Thus, unlike other PLP-dependent enzymes, for which significant changes in the tertiary structure associated with the apo-to-holo transition were reported [26, 27], the binding of PLP to apo-hOAT does not seem to alter the microenvironment of aromatic amino acids of the enzyme, but only induce a surface alteration of the protein, as suggested by the ANS emission fluorescence spectra.

### Quaternary Structure Analysis

**Fig. 2:**
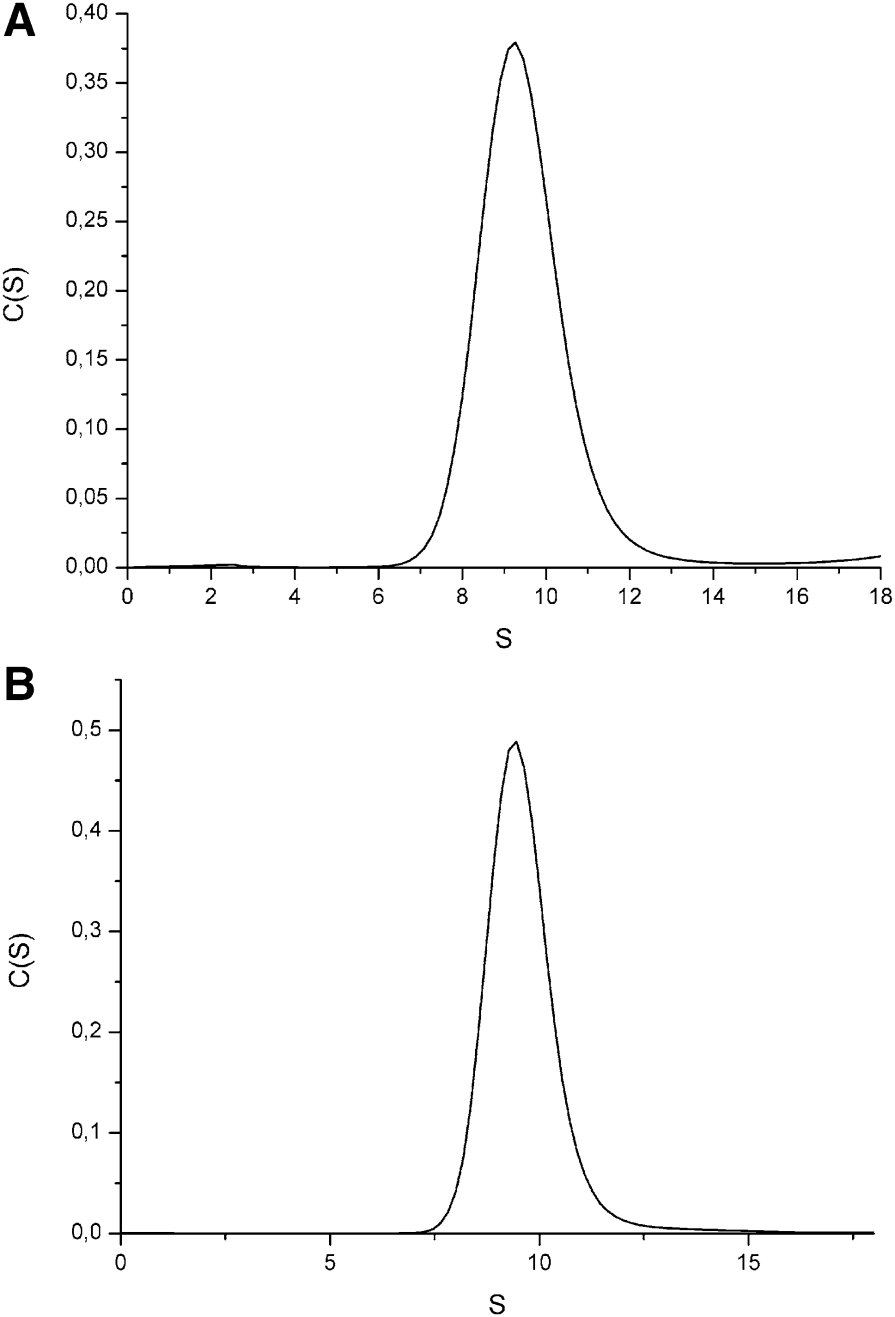
Sedimentation profile of hOAT. Sedimentation profile of holo (**a**) and apo (**b**) hOAT. The analysis were performed at 25,000 rpm, at 20 °C, at a protein concentration of 6 µM in the buffers 50 mM Hepes 0.5 M NaCl pH 7.4

**Fig. 3:**
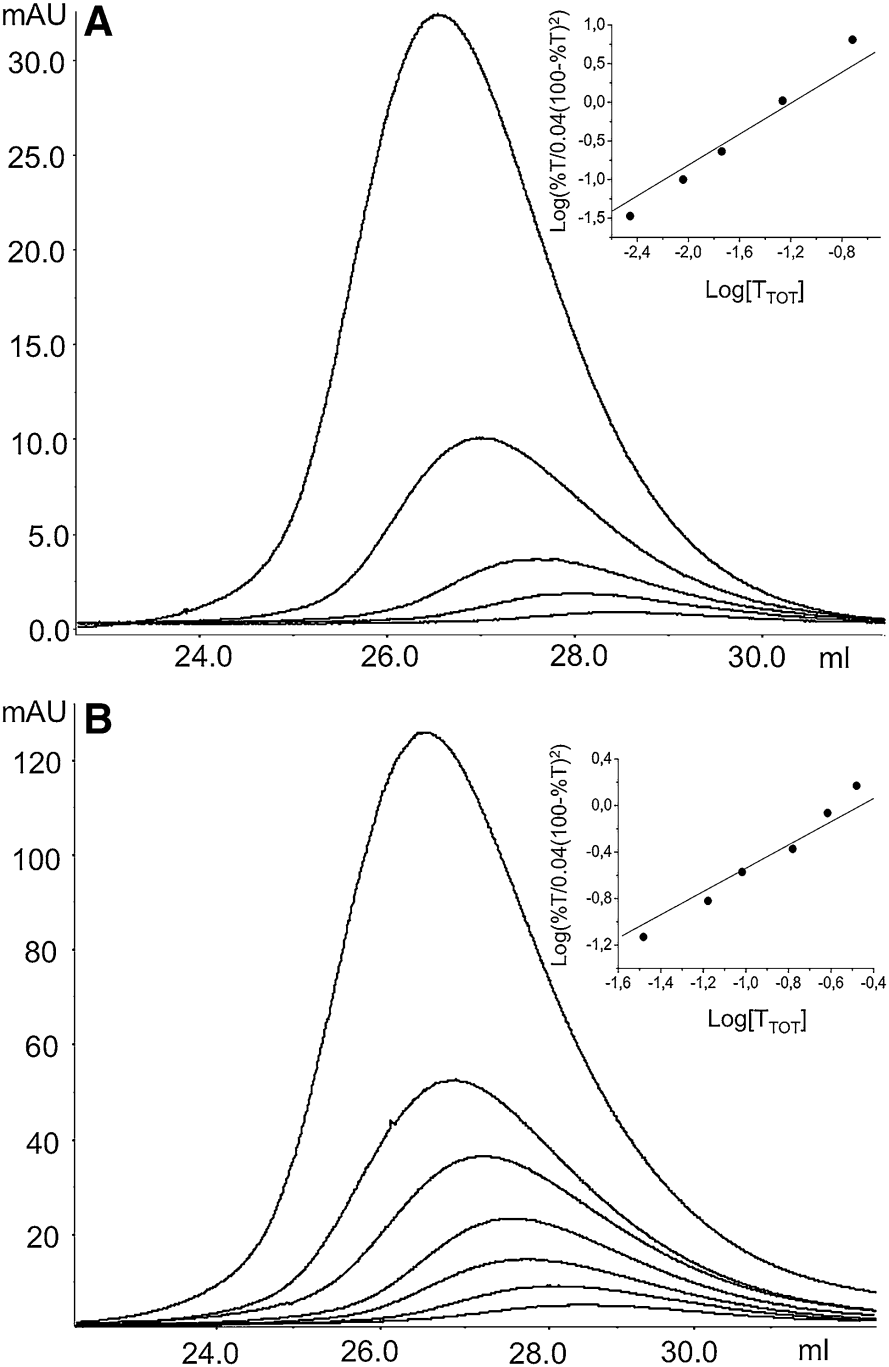
SEC analysis of wild-type hOAT. SEC profiles of holo (**a**) and apo (**b**) hOAT in the concentration range 0.3–15 and 3–75 μM respectively. A Sephacryl S300 column equilibrated in 50 mM Hepes, pH 7.4, 0.5 M NaCl was used. The *inset* of **a** and **b** show the plot of the log(%T/0.04 (100 − %T)^2^) vs. log[T_TOT_] of holo- and apo-hOAT, respectively

To investigate the hOAT oligomerization state, holo and apo-hOAT were analysed by AUC and SEC. Each sedimentation profile shows a unique peak with a sedimentation coefficient value of S20,W of 9.5 S for the holoprotein (Fig. 2a) and 9.6 S for the apoprotein (Fig. 2b). Such values correspond to a molecular weight of 183 kDa, a value nearly identical to that expected for a tetrameric species (183.4 kDa). The SEC analysis of holo and apo-hOAT was performed upon pre-incubation of both species at concentrations ranging from 0.15 to 75 μM for 20′ (a time long enough to guarantee the achievement of an equilibrium condition) at 25 °C. At the highest concentration both species eluted as a single peak characterized by a retention volume (Ve) of 26.2 and 26.5 mL for holo and apo-hOAT, respectively. On the basis of the calibration curve such values correspond to an apparent molecular weight of 182 kDa for holo-hOAT and 172 kDa for apo-hOAT. Thus, in agreement with AUC results, both species exhibit a molecular weight compatible with a tetrameric structure. Moreover, the Ve of both holo and apo-hOAT increases at decreasing enzyme concentrations down to a value corresponding to a dimeric species (28.8 mL) (Fig. 3a, b). This behavior indicates the presence of both dimeric and tetrameric hOAT in rapid equilibrium. Following the method of Manning et al. [25], the tetramer–dimer equilibrium dissociation constants (K_D(tet−dim)_) of holo-hOAT and apo-hOAT were found to be 0.070 ± 0.005 and 0.32 ± 0.04 μM (expressed as tetrameric unit), respectively. Accordingly, in DLS experiments, performed at 0.5 μM enzyme concentration, the hydrodynamic diameter of holo-hOAT resulted ~12 nm, while that of apo-hOAT was ~9.9 nm (data not shown). On the basis of the K_D(tet−dim)_ values, at 0.5 μM enzyme concentration holo-hOAT is almost completely in tetrameric form while only 75% of apo-hOAT is expected to be tetrameric.

### Computational Analysis of the Dimer–Dimer Interface

**Fig. 4:**
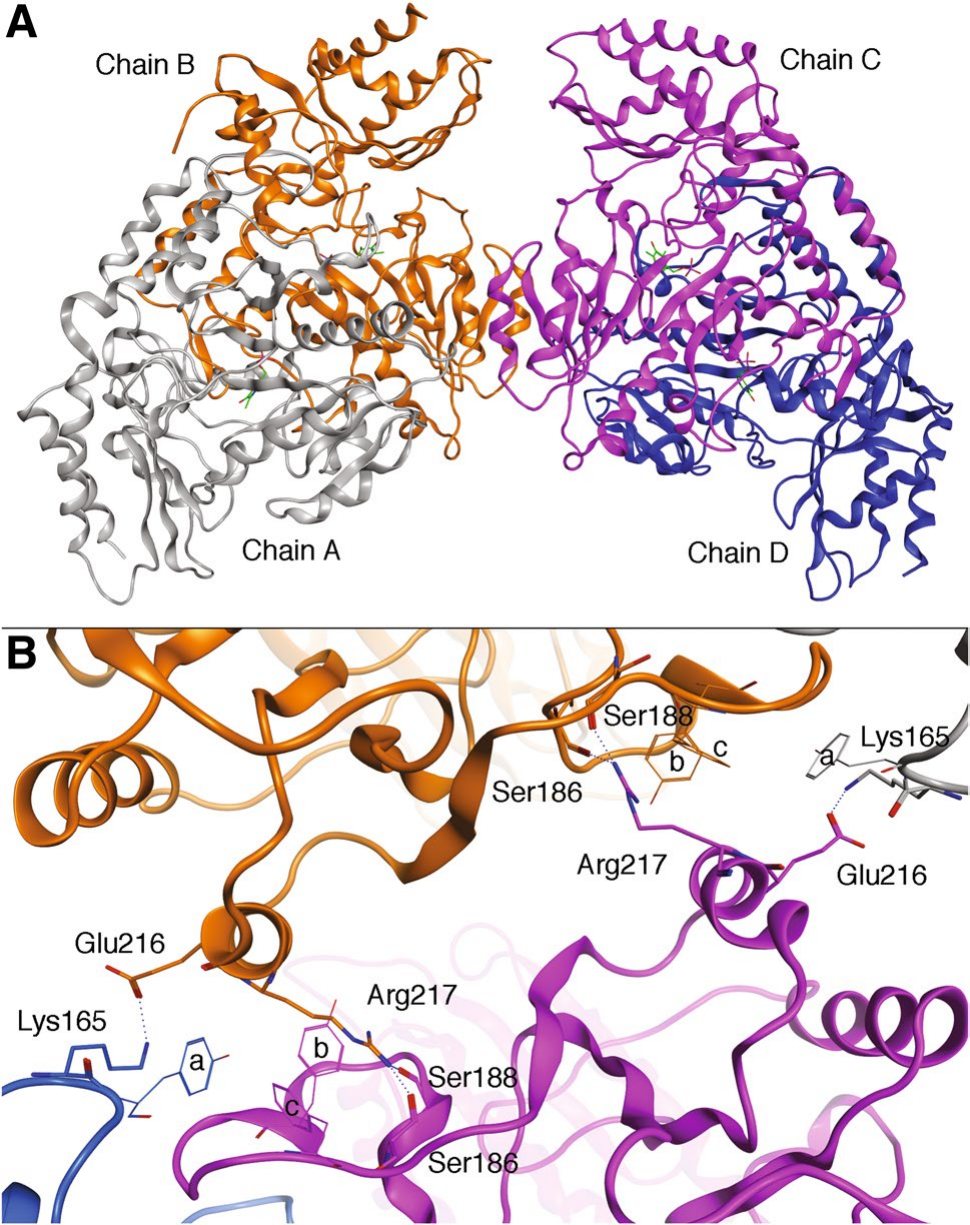
Dimer–dimer interface of the hOAT tetramer. **a** Ribbons representation of the hOAT tetramer. The chains of the two dimers are indicated and differently colored. *Green sticks* represent the PLP molecules. **b** Detail of inter-dimer interactions. Residues involved are indicated and represented as *sticks*. The side chains of Tyr166 (**a**), Tyr194 (**b**) and Phe200 (**c**) surrounding the Arg217 side chain are also represented. Image was rendered using the MOE software (CCG group). (Color figure online)

We decided to perform an “in silico” inspection of the dimer–dimer interface in a modelled tetrameric structure with the following aims: (a) define the structural determinants of hOAT tetramerization and (b) understand how PLP can stabilize hOAT tetramer. The solved crystal structure of hOAT (pdb file 1OAT) comprises one complete dimer (chain A and B in Fig. 4a) interacting with one monomer (chain C in Fig. 4a) belonging to the neighboring dimeric unit. Starting from the existing structures of the hOAT dimer and trimer we obtained a tetramer structure that was refined by energy minimization (“Materials and Methods” section). The model comprises a dimer–dimer interface (Fig. 4a) that was analyzed in order to map the inter-chain contacts. As expected, all contacts between the hOAT dimers are located quite distant from the PLP interaction network. The main cluster of inter-dimer contacts is supported by α-helix 8 of the chains B and C. In particular, Glu216 is in contact with the ε-amino group of Lys165* while Arg217 is in optimal position to form a H-bond with the backbone carbonyl groups of Ser186* or Ser188* (*denoting a residue belonging to the neighboring dimer). Moreover, the long apolar portion of the Arg217 side chain possibly contributes to dimer–dimer hydrophobic interactions, since it lies into a hydrophobic cleft composed by Phe200*, Tyr194* and Tyr166* (Fig. 4b). On the basis of the above observations on dimer–dimer interface of hOAT, we attempted to destabilize the tetrameric assembly. We focused our attention on Arg217, whose substitution to Ala is expected not only to abolish two polar contacts, but also to reduce the hydrophobic contact surface between dimers. Moreover, the amino acid conservation analysis generated by the Consurf server reveals that Arg217 is conserved in mammals (data not shown). On the basis of these bioinformatic analyses, the R217A variant was constructed by site directed mutagenesis, expressed and purified.

### Characterization of the R217A Variant

**Fig. 5:**
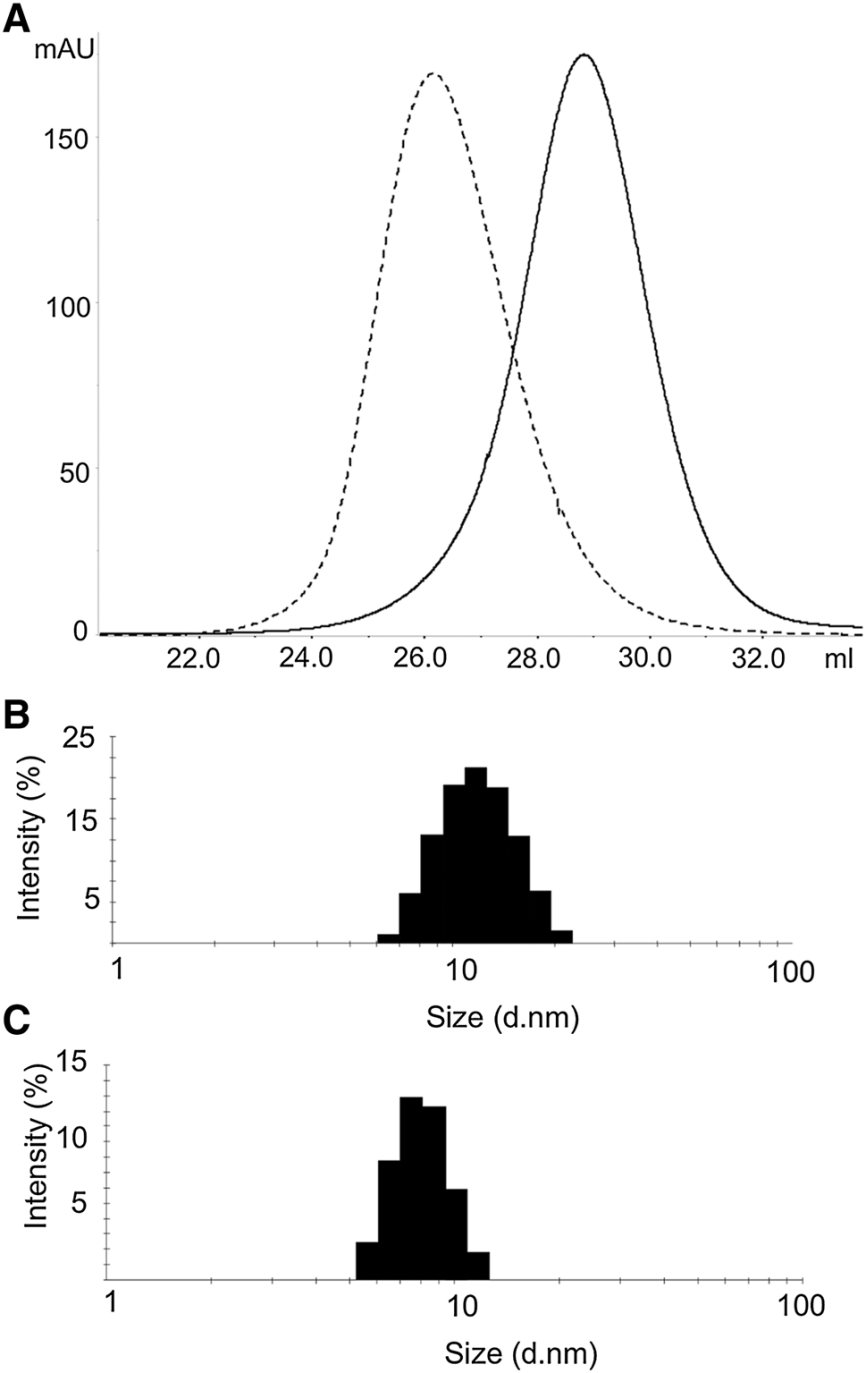
Molecular dimensions of the R217A variant. **a** SEC profile of wild-type hOAT (*dashed line*) and of the R217A (*normal line*) variant at 75 μM concentration on a Sephacryl S300 column. DLS analysis of the size distribution of 6 μM **b** hOAT wild-type and **c** R217A. Both analyses were performed in 50 mM Hepes, pH 7.4, 0.5 M NaCl, at 25 °C

The effect of the R217A mutation on the quaternary structure of hOAT was investigated by SEC analysis and DLS measurements. From 1 to 75 μM concentration, R217A elutes from SEC as a single symmetric peak with a Ve (29 mL) corresponding to a molecular weight of 88 kDa attributable to a dimeric species (Fig. 5a). DLS analyses indicate that R217A exhibits a mean molecular diameter of ~8 nm corresponding to a MW of 80–90 KDa, compatible with a dimeric assembly (Fig. 5b), and no changes of the molecular dimensions were observed in the presence of l-Orn and α-KG at saturating concentrations. Altogether, these results prove that the R217A variant is dimeric up to 75 μM concentration and maintains the dimeric structure upon substrate binding. Thus, it represents a good model to study the properties of dimeric hOAT. The absorbance, CD and fluorescence spectra of holo- and apo-R217A appear substantially identical to those of wild-type hOAT (Online Resource Fig. 1). Thus, the R217A substitution does not change the secondary and tertiary structure as well as the internal aldimine microenvironment of hOAT. The K_D(PLP)_ of R217A results to be 0.28 ± 0.3 μM, a value very similar to that of the wild-type. However, on the basis of the K_D(tet−dim)_ values, the K_D(PLP)_ of apo-hOAT wild type has been measured for a dimeric apoform which partly underwent tetramerization upon PLP binding. Unfortunately, we could not measure the K_D(PLP)_ value for the tetrameric apo-hOAT because of the high enzyme concentration required, which would prevent the establishment of equilibrium condition. Thus, we cannot compare the PLP binding affinities for dimeric and tetrameric apo-hOAT.

### Functional Properties of Wild-Type hOAT and of the R217A Variant

By determining the steady-state kinetic parameters of hOAT for the pair l-Orn/α-KG, we found that the Michaelis constant (K_M_) for l-Orn increases as the buffer ionic strength increases reaching a value of 38 mM if measured in Hepes 50 mM pH 7.4, 0.5 M NaCl. In order to better mimic the physiological ionic strength and pH conditions reported for the mitochondrial matrix [28] we measured the kinetic parameters in 50 mM Hepes, pH 8.0, 150 mM NaCl. At first both wild-type hOAT and R217A were assayed at 60 nM enzyme concentration at 25 °C. We verified that, under these conditions both species are present in solution mostly as dimers. For wild-type OAT we calculated K_M_ values of 6.5 ± 0.4 and 3.9 ± 0.5 mM for l-Orn and α-KG, respectively, and a *k*_cat_ of ~35 s^−1^ (Table 2). These values are comparable with those reported for hOAT purified from liver [15], and other mammalian aminotransferases [27, 29] as well as for OATs from plant [8, 9]. The steady state kinetic parameters of the R217A variant resulted very similar to those of wild type hOAT (Table 2), indicating that the mutation does not affect the catalytic activity of the enzyme and confirming that R217A variant represents a good model of the hOAT dimer. To investigate if the tetrameric structure could affect the kinetic parameters we performed the assays at 1 μM enzyme, a concentration at which the wild-type enzyme is mainly tetrameric whereas the R217A is dimeric. In order to slow down the rate of catalysis and to collect reliable data the assays were performed at 15 °C. Results shown in Table 3 indicate that tetrameric wild-type OAT and dimeric R217A share the same kinetic parameters and reveal that in hOAT the minimal functional unit is the dimer and not the tetramer. We could not determine the steady state kinetic parameters for the pair GSA:l-glutamate because GSA spontaneously cyclizes to P5C making the first half-reaction virtually irreversible.

**Table 2:** Kinetic parameters measured at 25 °C and 60 nM enzyme concentration

Enzymatic species	Substrate	Co-substrate	K_M(l−Orn)_ (mM)	K_M(α−KG)_ (mM)	*k*_cat_ (s^−1^)	*k*_cat_/K_M_ (M^−1^ s^−1^)
Wild-type	l-Orn	α-KG	6.5 ± 0.4	3.9 ± 0.5	34.9 ± 0.6	5.4 ± 0.3
	α-KG	l-Orn			35.7 ± 0.7	9.1 ± 1.2
R217A	l-Orn	α-KG	5.1 ± 0.3	2.4 ± 0.1	38.4 ± 0.7	7.5 ± 0.5
	α-KG	l-Orn				
					38.1 ± 0.3	15.9 ± 0.8

**Table 3:** Kinetic parameters measured at 15 °C and 1 μM enzyme concentration

Enzymatic species	Substrate	Co-substrate	K_M(l−Orn)_ (mM)	K_M(α−KG)_ (mM)	*k*_cat_ (s^−1^)	*k*_cat_/K_M_ (M^−1^ s^−1^)
Wild-type	l-Orn	α-KG	7.0 ± 0.5	4.0 ± 0.3	22.6 ± 0.5	3.2 ± 0.2
	α-KG	l-Orn			20.0 ± 0.3	5.0 ± 0.4
R217A	l-Orn	α-KG	6.9 ± 0.4	4.4 ± 0.3	20.7 ± 0.4	2.2 ± 0.1
	α-KG	l-Orn			20.7 ± 0.3	4.7 ± 0.3

### Thermal Stability and Aggregation Propensity of hOAT

**Fig. 6:**
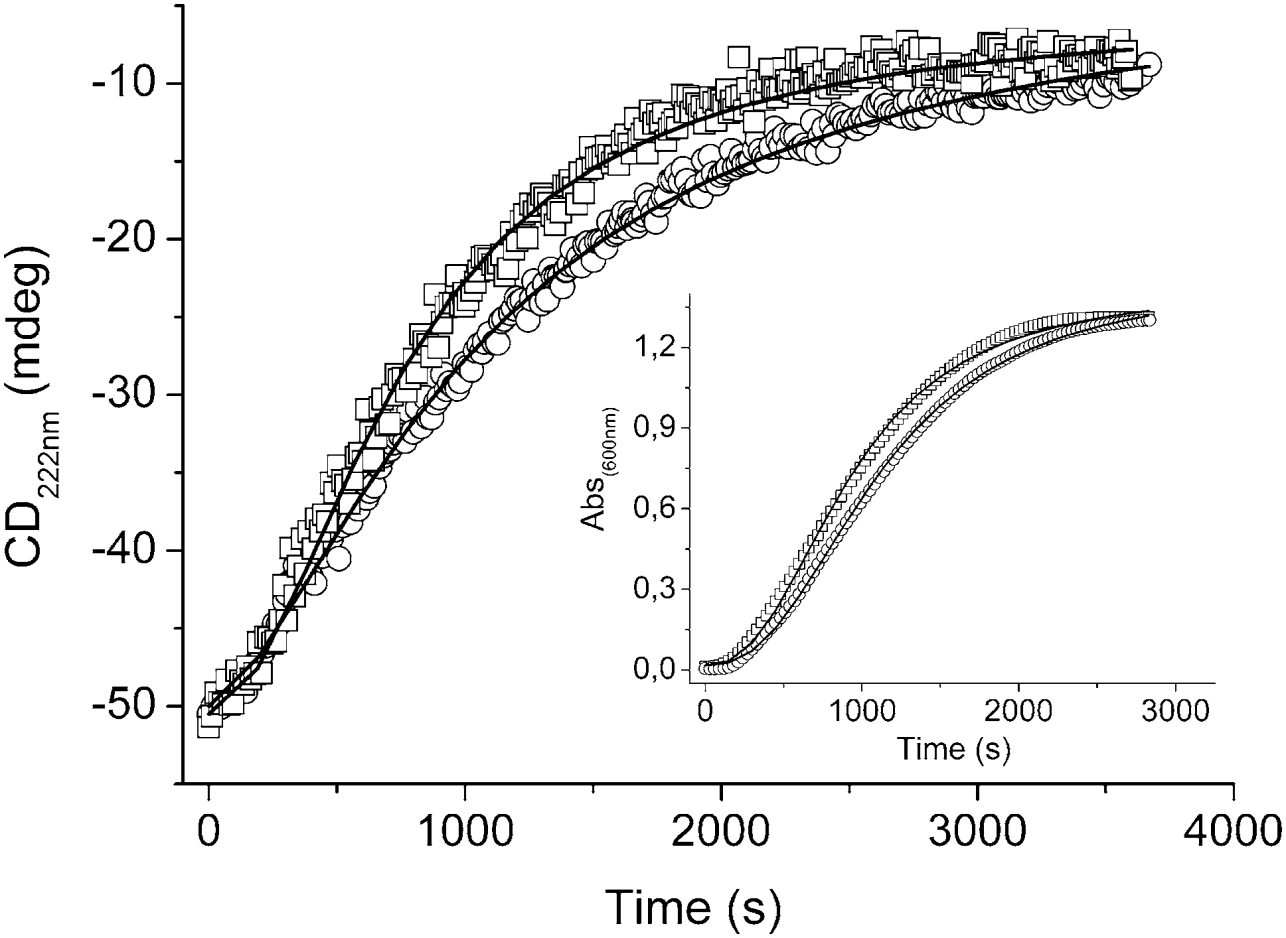
Unfolding and aggregation curves of apo-hOAT wild-type and apo-R217A variant under physiological conditions. Trends of the CD signal at 222 nm and of the absorbance at 600 nm (*inset*) during the incubation of 6 μM apo-hOAT wild-type (*open circle*) and apo-R217A (*open square*) at 37 °C in PBS pH 8.0. Trends were fitted to a logistic curve by Origin7 software (OriginLab)

To investigate if PLP could affect the thermal stability of the enzyme we determined the melting temperature (T_m_) of holo and apo-hOAT in the tetrameric form following the decrease of the dichroic signal at 222 and 422 nm, which indicate of the loss of the enzyme secondary structure and of the bound PLP, respectively (Table 4). Holo-hOAT denatured and released PLP in a single-step concerted process with apparent melting temperatures of 67.0 ± 0.1 and 66.8 ± 0.1 °C, respectively. In contrast, apo-hOAT showed an apparent T_m_ value of 46.1 ± 0.1 °C, a value ~21 °C lower than that of the holo-form. As a model of dimeric OAT, we determined the apparent T_m_ values of both the apo- and holo-R217A variant, which resulted similar to the corresponding ones of the wild-type enzyme (Table 4). These data indicate that tetrameric and dimeric holo-hOAT share a similar resistance to thermal stress which is much higher than that of apo-hOAT both in dimeric and tetrameric form. Thus, the apo-to-holo transition is associated with a significant increase of the protein thermal stability. Since the T_m_ value of apo-hOAT results quite near to the physiological temperature, we examined the susceptibility to unfolding and aggregation under physiological conditions of wild-type OAT and of the R217A variant in both apo- and holo-form. To this purpose we monitored the CD signal at 222 nm and the absorbance at 600 nm during the incubation of 6 μM enzyme in PBS buffer pH 8.0 at 37 °C. While no changes could be detected for both holo-forms up to 2 h incubation, both apo-forms showed significant changes of the 222 nm CD signal and of turbidity reflecting the loss of the secondary structure and an ongoing aggregation process, respectively (Fig. 6). The half time (t_1/2_) of the process was determined by fitting the signal changes to a logistic curve. For apo-hOAT wild-type, t_1/2_ of 17.9 ± 0.1 and 19.6 ± 0.2 min were calculated for the unfolding and the aggregation processes, respectively. The two values are in good agreement, revealing that under physiological conditions apo-hOAT wild-type undergoes a rapid unfolding process accompanied by aggregation. A qualitatively similar behavior was observed for apo-R217A (Fig. 6). However, the t_1/2_ for the unfolding and aggregation processes resulted to be 12.7 ± 0.2 and 14.7 ± 0.1 min, respectively. Considering that at 6 μM enzyme concentration apo-hOAT wild-type is mainly tetrameric while the mutant form is completely dimeric, we could conclude that the tetrameric state only slightly enhances the stability of apo-hOAT by increasing the halftime of unfolding of ~1.3–1.4 fold.

**Table 4:** Melting temperatures of wild-type OAT and R217A variant

Enzymatic species	T_m holo (222 nm)_ (°C)	T_m holo (422 nm)_ (°C)	T_m apo (222 nm)_ (°C)
Wild-type	67.0 ± 0.1	68.6 ± 0.1	45.8 ± 0.1
R217A	68.7 ± 0.1	67.4 ± 0.1	44.3 ± 0.1

## Discussion

The comparison between the biochemical properties of the apo- and the holo-forms of hOAT was performed under experimental conditions (50 mM Hepes, 500 mM NaCl, pH 7.4) in which both forms are soluble. The results reveal that apo and holo-hOAT exhibit similar tertiary structure, as shown by their near-UV CD spectra. Changes of the dichroic bands in the near-UV region during the apo to holo transition in Fold Type I aminotransferases were previously associated with changes in the position/orientation of aromatic residues at or in proximity of the active site [33]. In this family, PLP coordination typically involves a planar π base stacking interaction between the PLP pyridine ring and a tryptophan indolic ring, along with an H-bond interaction between a tyrosine residue belonging to the neighboring subunit and the phosphate group of PLP. The inspection of the hOAT active site (Online Resource Fig. 2) shows that PLP forms a π–π hydrophobic interaction with the phenyl ring of Phe177, which is oriented perpendicularly with respect to the pyridine ring, and that the phosphate group forms two hydrogen bonds with the peptidic N of Val143 and with the side chain of Thr322* of the neighboring subunit. The finding that the typical interactions between PLP and aromatic residues are not present in the hOAT coenzyme binding pocket could explain the minimal impact of PLP binding on the dichroic features in the near-UV region of the protein. Nonetheless, the apo-form of hOAT exhibits an exposure of hydrophobic surfaces to the solvent more pronounced than the holo-form, thus suggesting that PLP binding could affect protein conformation. This view is supported by the different apparent T_m_ value of holo-(67 °C) and apo-hOAT (46 °C). Moreover, we demonstrated that both holo and apo exist in solution as a tetramer. Although these data confirm previous analyses on mammalian OATs purified from different sources [13–16], they are in contrast with crystallographic analyses indicating the hexameric assembly of the enzyme [18–21]. It is possible that the crystallization conditions, known to increase the solute concentration over that expected for an ideal solution, could promote the hexamerization. Anyway, it is of interest to note that apo-hOAT exhibits a K_D(tet−dim)_ value fivefold higher that of holo-hOAT, thus indicating that PLP binding plays a role in shifting the equilibrium toward the tetrameric species. The ability of PLP to promote the dimerization of enzymes belonging to the fold type I class is well known [30–32]. This is not surprising considering that in this class of enzymes the dimer is the functional unit as each active site is made of amino acid residues provided by both monomers in the dimer. The modeled tetrameric structure of hOAT allowed us to establish that the active site is located far from the tetramer interface. Thus, at present, it is not easy to understand how PLP binding can impact the tetrameric structure of the enzyme. In this regard, a conformational change has been associated to the PLP-induced tetramerization of mitochondrial human serine hydroxymethyltransferase [33]. The authors have suggested that PLP binding to the apoform shifts the equilibrium from an “open” to a “close” conformation of the dimers promoting the tetramerization. Disappointingly, the absence of the apo-hOAT crystal structure makes difficult to identify structural alterations of hOAT induced by PLP-binding. We can only envisage that remote effects of PLP binding on the position of Ser186 and Ser188 could be transmitted throughout the segment 178–188 starting from Phe177 which forms a π–π interaction with the PLP pyridine ring (Online Resource Fig. 3). Additionally, the visual inspection of the tetrameric structure allowed us to identify Arg217 as a hot-spot at the dimer–dimer interface. Therefore, an artificial R217A mutant was constructed, cloned, purified and characterized. The unexpected finding that the mutant form displays a dimeric structure endowed with spectroscopic and catalytic features as well as apparent T_m_ values similar to the corresponding ones of the tetrameric form of hOAT demonstrate that the functional unit of the enzyme is a dimer and not a tetramer. On this basis, we decided to compare the apo- and holo-form of the tetramer with those of the dimer under physiological conditions. The results reveal that, unlike the holo-tetramer and dimer, both apo-tetramer and apo-dimer undergo a rapid unfolding process accompanied by aggregation, even if these events appear slightly more pronounced for the apo-tetramer than for the apo-dimer. Thus, it can be concluded that the holo- and apo-hOAT have different conformations under physiological conditions. A reduced stability of the apo-form with respect to the holo-form was reported for other PLP-dependent enzymes both “in vitro” and “in vivo” [34–36]. In some cases such behavior has been associated to differences in the global conformation and/or in the exposure of flexible regions [37]. To define the structural features at the basis of apo-hOAT instability the resolution of its crystal structure would be desirable. It can only be inferred from our results that the main determinant of the hOAT thermal stability under physiological conditions seems to be the binding of the coenzyme rather than the oligomeric state. It is reasonable that PLP concentration could greatly affect the fate of the enzyme, by increasing its thermal stability at physiological temperature and promoting the availability of folded OAT. Following this view, the coenzyme could represent not only a prosthetic group for hOAT, but also a key structural element affecting the OAT turnover. Further experiments will be necessary to define if and how the presence of PLP could influence the stability and the tetramerization of hOAT “in vitro” and/or in a cellular environment.

## Electronic supplementary material

Below is the link to the electronic supplementary material.

Supplementary material 1 (PDF 608 KB)

## Abbreviations

hOAT: Human ornithine aminotransferase
PLP: Pyridoxal 5′-phosphate
P5C: Pirroline-5-carboxylate
l-Orn: l-Ornithine
α-KG: α-Ketoglutarate
AUC: Analytical ultracentrifugation
K_D(tet−dim)_: Tetramer–dimer equilibrium dissociation constant
K_D(PLP)_: Equilibrium dissociation constant for PLP
