# Supplementary material for: Oligomeric State and Thermal Stability of Apo- and Holo- Human Ornithine δ-Aminotransferase
Source: Protein J. 2017 Mar 27;36(3):174–85. doi: 10.1007/s10930-017-9710-5 (PMC5432616; doi:10.1007/s10930-017-9710-5)
Supplement: Supplementary file 1 — Supplementary material 1 (PDF 608 KB) [file 10930_2017_9710_MOESM1_ESM.pdf]

OLIGOMERIC STATE AND THERMAL STABILITY OF APO- AND HOLO- HUMAN ORNITHINE δ-AMINOTRANSFERASE

The Protein Journal

Riccardo Montioli[1*], Carlotta Zamparelli[2], Carla Borri Voltattorni[1] and Barbara Cellini[1]

[1]Department of Neuroscience, Biomedicine and Movement sciences (Section of Biological Chemistry), University of Verona, Verona Italy.

[2]Department of Biochemical Sciences, University "La Sapienza", Rome, Italy

*To whom correspondence should be addressed at: Department of Neurosciences, Biomedicine and Movement sciences (Section of Biological Chemistry) Strada Le Grazie 8, 37134 Verona, Italy. E-mail: riccardo.montioli@univr.it

Supplementary figures

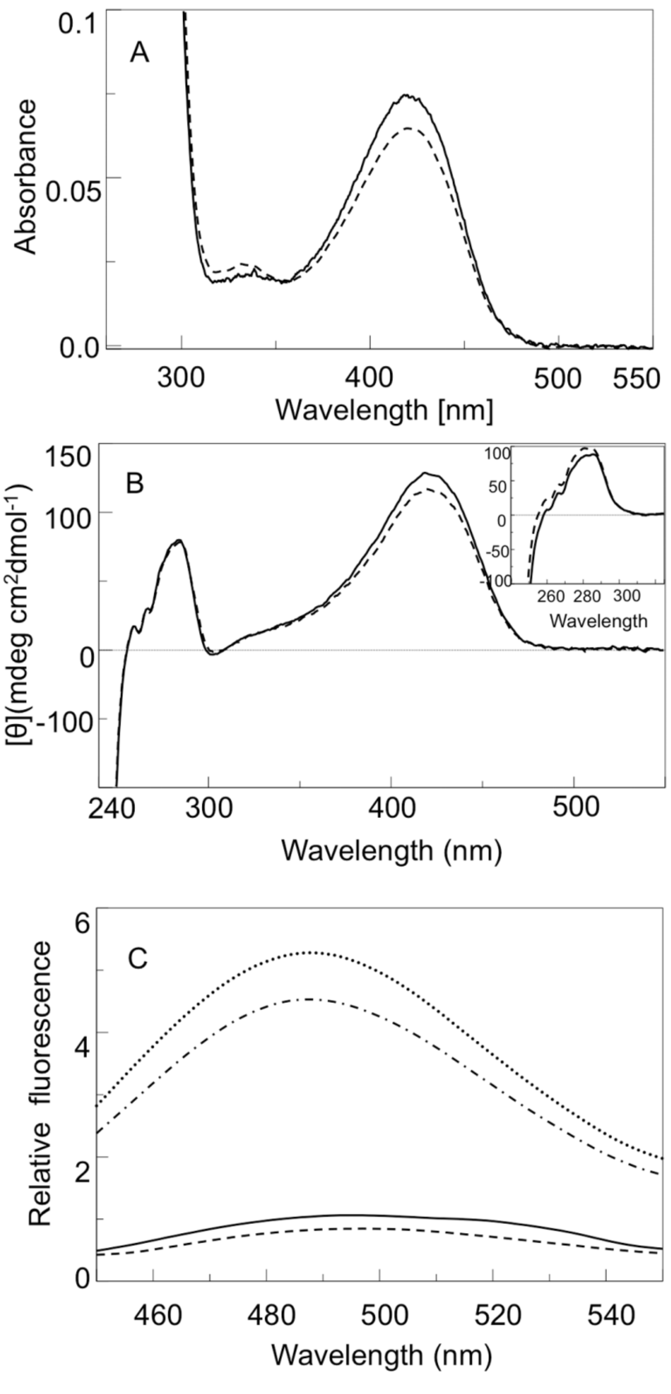

Fig. 1. Spectroscopic features of R217A mutant. (A) absorbance and (B) near UV-Vis CD spectra of 6 µM holo-hOAT wild type (----) and holo-R217A (—); (inset panel B) near UV CD spectra of 6 µM apo-hOAT wild type (----) and apo-R217A (—). (C) ANS fluorescence of 6 µM apo-hOAT wild type (·-·-), apo-R217A (·····), holo-hOAT wild type (----) and holo-R217A (—). All the spectra were registered in 50 mM Hepes buffer, pH 7.4, 0.5 M NaCl.

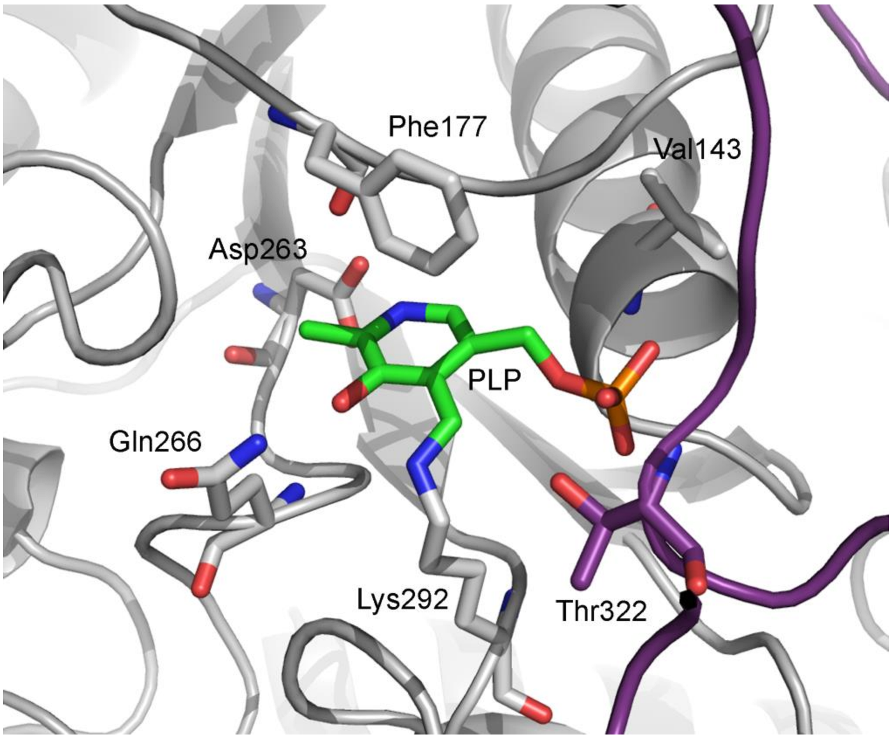

Fig. 2. OAT active site. Ribbons representation of the OAT active site in which the two monomers are colored white and purple, respectively. PLP is represented as green sticks and the residues contributing to PLP binding are indicated.

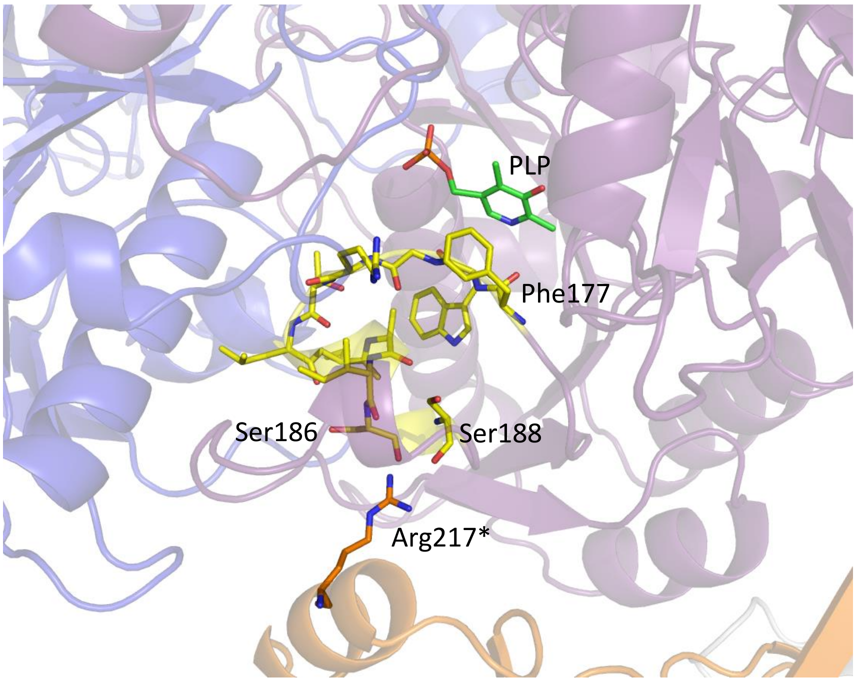

Fig. 3. Representation of the segment 177-188. Sticks representation of the segment 177-188 (yellow). The positions of Phe177, Ser186 and Ser188 are indicated. Arg217* and the PLP molecule are represented as orange and green sticks respectively (*denoting a residue belonging to the neighboring dimer). The image was rendered using PyMol software (Schrödinger).
